# Gas-phase molybdenum-99 separation from uranium dioxide by fluoride volatility using nitrogen trifluoride

**DOI:** 10.1039/c9ra10270a

**Published:** 2020-01-21

**Authors:** Bruce K. McNamara, Matthew J. O'Hara, Richard A. Clark, Samuel S. Morrison, Chuck Z. Soderquist, Randall D. Scheele

**Affiliations:** Pacific Northwest National Laboratory PO Box 902, Battelle Blvd Richland Wa 99352 USA bruce.mcnamara@pnnl.gov; Flibe Energy Richland Wa 99352 USA

## Abstract

Production of the important ^99m^Tc medical isotope parent, molybdenum-99 (^99^Mo), *via* the fissioning of high- and low-enriched uranium (HEU/LEU) targets followed by target dissolution in acid and solution-phase purification of ^99^Mo is time-consuming, generates quantities of corrosive radioactive waste, and can result in the release of an array of radionuclides to the atmosphere. An alternative ^99^Mo purification method has been devised that has the potential to alleviate many of these issues. Herein, we demonstrate the feasibility of a rapid Mo/Tc gas-phase separation from UO_2_. The results indicate that volatile [^99^Mo]Mo can be captured downstream of the reacted solid mixture on a column bed (trap) of alumina; the majority of the captured [^99^Mo]Mo can be subsequently eluted from the alumina trap with a few milliliters of water. >1.0 × 10^5^ single pass decontamination of U and the collected [^99^Mo]Mo product is demonstrated. This simple thermo-fluorination technique has the potential to provide a rapid methodology for routine ^99^Mo production.

## Abstract 2

Data demonstrates large variance in the volatilization temperatures of MoO_2_ (likely as MoF_6_) *vs.* UO_2_ (as UF_6_). This forms the basis of a gas-phase separation of ^99^Mo from U.

## Graphical abstract

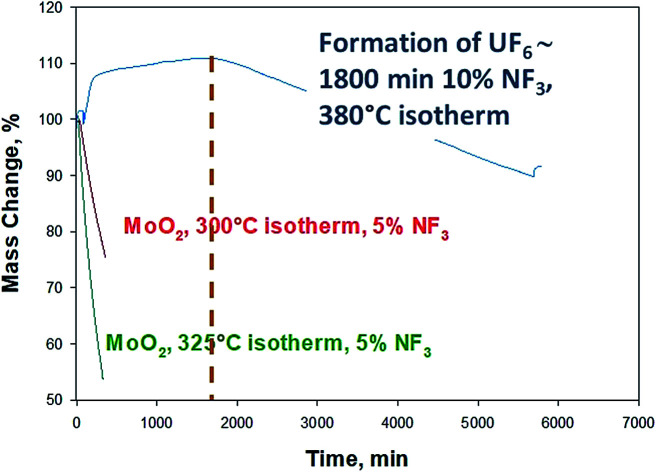

## 1. Introduction

Technetium-99m (^99m^Tc, *t*_1/2_ = 6.01 h) is the most widely used diagnostic radionuclide world-wide. It is dispensed at radiopharmacies and hospitals *via*^99^Mo/^99m^Tc generators.^1^ The ^99^Mo (*t*_1/2_ = 65.98 h, ∼6.1% fission yield) parent is produced *via* the fissioning of highly enriched uranium (HEU) targets. The ^99^Mo is chemically purified from the target material and other fission and activation products by processing of the acid-dissolved HEU targets.^2^ Processing of the dissolved HEU targets requires several days, produces corrosive liquid waste streams, and an enriched uranium waste stream. Further issues related to chemical processing for planned conversions of HEU target materials to LEU targets have been discussed by Vandergrift and other researchers.^2,3^

In this article, we discuss the volatility profiles and separation protocol for the ^99^Mo/^99m^Tc couple from uranium using nitrogen trifluoride. In most of the proposed fluorination methods such as the FLUOREX process,^4^ or those described earlier by workers in the Czech Republic,^5^ the former Soviet Union,^6^ or the US,^7–9^ fluorine gas is used to rapidly form volatile UF_6_ from an irradiated matrix, generally UO_2_ or U metal^10^ A large set of volatile fluoride products such as PuF_6_, NpF_6_, IF_*x*_ (for *x* = 3, 5, 7), TeF_6_, TcF_6_, *etc.*, can be binned cryogenically, or can be sorbed onto solid traps that have specific capture affinities for the volatile products.^10,11^ Regardless of the exact process used, fluorination requires the use of rigorously closed reactor and trapping systems. These systems are thus more suited to complete trapping of volatile fission products than the liquid digestion processes currently in use.

The usefulness of NF_3_ for volatility separations is related to its slightly lower thermal reactivity compared to more potent fluorinating reagents. The lower reactivity of NF_3_ allows for volatility of a reduced set of fission products, in particular, Mo and Tc, without volatilization of U, Np and Pu. The basis for the separations is the formation of thermally stable, nonvolatile UO_2_F_2_ produced as the first product in the fluorination of UO_2_ (eqn (1)), or of UF_4_ in the case of the U metal fluorination (eqn (2)):1
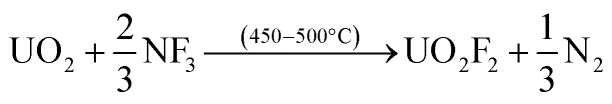
2
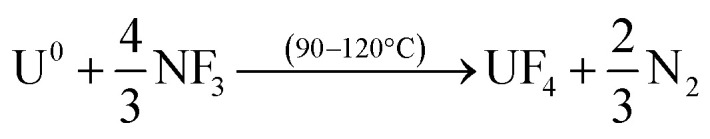


The fluorinated solid matrix formed per eqn (1) or (2) can be further reacted with NF_3_ to extract volatile fluorides without formation of gaseous UF_6_. The onset temperature for the conversion of UO_2_F_2_ to UF_6_ is usually near 500 °C, but can be stalled nearly completely by lowering the NF_3_ concentration to 1 or 5% in Ar.^12^ Similarly, in a metal target the conversion of UF_6_ from UF_4_ can be considerably slowed using reduced temperature or lower NF_3_ concentrations.^13^ This feature of the reactions allows for gaseous leaching of the solid U-bearing sample for the time required to volatilize lower boiling point components (such as ^99^Mo/^99m^Tc) that are generally shown to be rapidly separated at or below 400 °C. Separation and recovery of volatile MoF_6_/TcF_6_ from other fission products has been demonstrated by use of selective sorbents, such as solid MgF_2_.^14^

High temperature oxidation of irradiated U has been widely cited as being effective at removal of gaseous fission products such as Xe and Kr.^15^ This is more rapidly and completely realized by the lattice disruption of the U solid, as induced by fluorination (eqn (2) and (3)). Fluorination using NF_3_ will volatilize Nb, Mo, Sb, Tc, Te^16,17^ and I from a solid matrix at or below ∼400 °C. Ru will be released near 500 °C.^18^ Rhodium, Pd^18^ and Pu^17^ do not form volatile fluorides using NF_3_ as the fluorination reagent. Americium,^19^ the lanthanides, and the Group I and II elements do not form volatile fluorides using any fluorinating reagent.^20^

After down-stream capture of the volatile fission products has been performed, uranium can be recovered as gaseous UF_6_ per eqn (3) or (4), leaving the lanthanides, Pu, Am, and the other non-volatiles in the reactor furnace.3
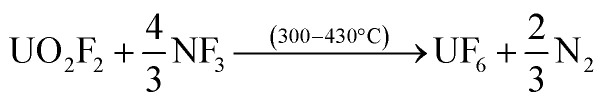
4
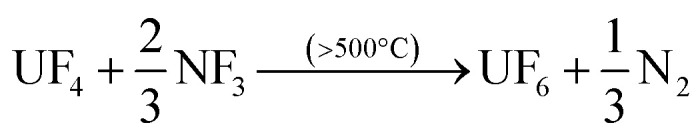
Here, we show that a gas/solid leaching process using NF_3_ to recover ^99^Mo/^99m^Tc from a simulated UO_2_ target has a sound empirical basis that promises rapid, single pass, high yield recovery of ^99^Mo/^99m^Tc.

## 2. Experimental

### 2.1 Reagents and materials

Mo metal, MoO_2_ and MoO_3_ were purchased from Alfa Aesar (Haverville, MA). Deionized water from a Barnstead E-Pure (Thermo Fisher, Waltham, MA) water purification system was 18.0 MΩ cm. Activated alumina spheres (0.125 inch dia.) were purchased from Delta Adsorbents (Roselle, IL). The sample and reference pans used in the TGA/DT and thermo-fluorination reactor were pressed in-house from 99.999% nickel, as 0.254 mm thick sheet purchased from EPSI Metals (Ashland, OR) and were preconditioned by treatment with NF_3_ up to 610 °C. Monel screens (400 mesh) were purchased from Cleveland Wire Cloth & Mfg. Co (Cleveland, OH).

Technetium-99 dioxide (^99^TcO_2_) was freshly prepared by thermal decomposition of NH_4_TcO_4_ (ref. 21) from house stocks at PNNL. Technetium-99 metal was prepared by heating ^99^TcO_2_ in a thermo-gravimetric furnace in a gas stream of 4% H_2_/Ar at 600 °C. The resulting ^99^Tc metal was a silver granular material. The metal was used in fluorination experiments immediately after each preparation.

For the mixed [^99^Mo]MoO_2_/UO_2_ experiment, sodium molybdate and sodium borohydride were purchased from Sigma-Aldrich (St. Louis, MO) and used as received. No-carrier-added (NCA) ^99^Mo/^99m^Tc solution in physiological saline solution was used as received from a commercial medical isotope supplier. A depleted uranium dioxide powder source from AREVA (Richland, WA) was used for the [^99^Mo]Mo/UO_2_ experiment.

### 2.2 [^99^Mo]MoO_2_/UO_2_ sample preparation

A homogeneous mixture of fine UO_2_ and [^99^Mo]MoO_2_ crystals, in a ∼7.5 : 1 mole ratio, was prepared in a nickel pan. The sample was prepared *via* the following steps, with Table 1 summarizing the reagent inputs: sodium molybdate (Na_2_MoO_4_) salt (2.35 mg) was added to a microcentrifuge tube (“tube 1”). A 0.76 mL aliquot of NCA ^99^Mo (79.9 ± 2.3 kBq, equivalent to 4.50 ± 0.13 pg) was added to the tube. The Na_2_MoO_4_ salts were allowed to completely dissolve and equilibrate in the tube, thus creating a homogeneous mixture of [^99^Mo]MoO_4_^2−^ ions. In a separate microcentrifuge tube (“tube 2”), NaBH_4_ salts (12.43 mg) were added and 0.2 mL H_2_O was used to dissolve the salts. The resulting NaBH_4_ solution was added to tube 1 and the solutions were mixed thoroughly.

**Table 1:** Reagent inputs for the formation of a homogeneous [^99^Mo]MoO_2_/UO_2_ mixture

Reagent	Reagent mass, mg	Mass ratio, reagent: Na_2_MoO_4_	Moles reagent	Mole ratio, reagent: [^99^Mo]MoO_2_
Na_2_MoO_4_	2.35^a^	—	1.14 × 10^−5^^b^	—
NaBH_4_	12.43	5.29	3.29 × 10^−4^	28.8
UO_2_	23.05	9.81	8.54 × 10^−5^	7.48

a Dissolved salts spiked to 34.0 ± 1.0 kBq ^99^Mo/mg Na_2_MoO_4_.

b Equivalent to moles [^99^Mo]MoO_2_ reaction product.

Precipitates of [^99^Mo]MoO_2_ began to form quickly in the presence of the reducing agent. After several hours, it was determined that the Mo(vi) → Mo(iv) reduction was complete. Tube 1 was centrifuged at ∼8000 rpm using Sorvall MC 12V centrifuge (Dupont, Newtown, CT). Next, the supernate was removed. Water (1 mL) was added to tube 1, and the [^99^Mo]MoO_2_ crystals were re-suspended. Depleted uranium dioxide powder (23.05 mg) was added to the tube and the [^99^Mo]MoO_2_/UO_2_ mixture was thoroughly mixed by sonication. Tube 1 was again centrifuged and the supernate discarded. The mixture was re-suspended in ∼250 μL water, and 50 μL aliquots of the suspension were added to a Ni sample pan that had been placed under an infrared heat lamp. The solid suspension was quantitatively added to the pan in successive ∼50 μL aliquots as the liquid in the pan was evaporated. Once thoroughly dried, the Ni pan containing the mixture of [^99^Mo]MoO_2_ and UO_2_ was transferred to a thermo-fluorination apparatus for gas-phase [^99^Mo]Mo separation from UO_2_.

### 2.3 Thermo-fluorination apparatus

**Fig. 1:**
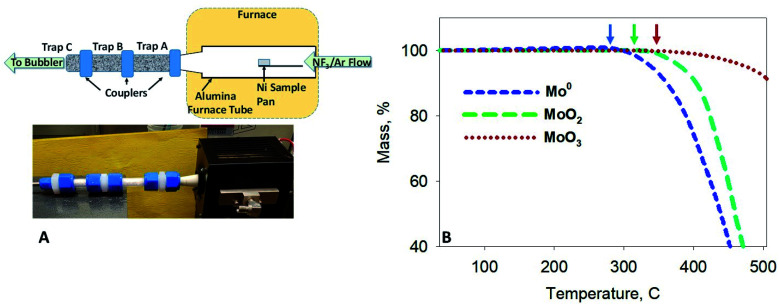
(A) Schematic and image of the fluorination apparatus. Two packed alumina traps (Traps A and B) and a quartz wool (Trap C) were coupled to the furnace tube outlet. Effluent gases were scrubbed through a bubbler trap prior to release. (B) Thermogravimetric scans of Mo metal, MoO_2_, and MoO_3_ powders exposed to a 5% NF_3_/Ar gas mixture. Arrows indicate the onset of volatility below 350 °C.

Thermogravimetric (TG) and differential thermal (DTA) screening data for the reaction of NF_3_ on samples of UO_2_, Mo and Tc metal, MoO_2_ and TcO_2_, and MoO_3_ (Fig. 1B) was acquired using a modified Seiko TG/DTA 320.^12^ The gases used for thermoanalytical experiments were 99.995% purity NF_3_ from Advanced Specialty Gases (Reno, NV) and 99.9995% ultra-high purity (UHP) Ar from OXARC (Pasco, WA). The same instrument was used in the reduction of ^99^TcO_2_ to ^99^Tc metal, wherein a stream of 4% H_2_ (99.99%) (OXARC) in Ar was used at 600 °C for 1 h.

Modification of the TG/DTA system included conditions for adequate gas mixing and improvements for corrosion resistance. NF_3_/UHP Ar gas mixtures were premixed in 4 linear feet of SS tubing (0.25 inch OD) prior to their entry into the furnace chamber of the TG apparatus. The premixed gas was routed through the analytical microbalance chamber by a 1/16 inch OD nickel tube to an area about 2.54 cm from the sample and reference pans. This distance reduced buoyancy motion of the sample and reference arms as the dense gas mixture was released from the nickel tube and also allowed for some laminar flow of the gas mixture along the direction of the sample. A larger UHP Ar flow was passed though the analytical balance and sensitive electronic components to protect them from a backflow of hot NF_3_ and other reaction product gases. Three flow meters were used to adjust the NF_3_/Ar concentration to a total gas flow rate of 200 mL min^−1^. The platinum thermocouples inside the balance beams were plated with nickel and the plating was covered in ceramic. The coatings help to reduce hot NF_3_ corrosion of the thermocouples for extended reaction screening of Mo, Tc and U samples, below 550 °C. The coatings were supplied by RT Instruments (Woodland, CA).

### 2.4 Fluorination protocol for [^99^Mo]MoO_2_/UO_2_ samples

The thermogravimetric apparatus described above was used to react the homogeneous [^99^Mo]MoO_2_/UO_2_ mixture with NF_3_. Two sequential activated alumina traps were attached to the 0.75 inch output of the TG alumina furnace tube as shown in Fig. 1A. The traps were made of 0.75 inch o.d. Teflon® PFA tubing (McMaster-Carr, Chicago, IL) assembled with Swagelok (Solon, OH) Teflon® PFA tube unions. The activated alumina spheres were contained in the tubing with the use of Monel screens placed within the tube unions; the spheres were slightly crushed to produce trap packing media that allowed unimpeded flow of gases. Behind the rear trap, a quartz wool plug was placed after the rear Monel screen. From there, a Teflon tube routed effluent gases through a 125 mL Erlenmeyer flask configured as a water bubbler.

At the end of a thermo-fluorination experiment, the traps and the furnace tube were disassembled, and each component was washed using a series of solvent washes as is described in detail below. The residual components in the nickel sample pan were fully analyzed by dissolution of the entire sample pan in nitric acid. The distribution of ^99^Mo was evaluated by gamma counting, and that of the U was evaluated by inductively coupled plasma-mass spectrometry (ICP-MS).

### 2.5 Radiometric measurements

1. HPGe: reference standards were prepared by spiking known volumes of ^99^Mo-bearing solutions (in secular equilibrium with ^99m^Tc) into 2.0 mL of 0.1 M HCl in 20 mL glass scintillation vials. These samples were analyzed using several high purity germanium (HPGe) gamma detectors (Ortec, Oak Ridge, TN) that had been energy and efficiency calibrated for this geometry using NIST traceable standards. Gamma spectra were evaluated using Genie 2000 Gamma Acquisition and Analysis software (v. 3.4.1) (Canberra, Meriden, CT). The mean ^99^Mo activity obtained in the reference standards using the HPGe detector analysis was used to establish the various detection efficiencies (*E*_d_) for ^99^Mo-bearing samples of non-standardized geometries using NaI(Tl) scintillation detectors (described below).

2. Auto-gamma counter: aqueous samples were prepared as 2.0 mL aliquots in 12 × 74 mm test tubes for counting on a Wizard 1470 (PerkinElmer, Meriden, CT) automatic gamma counter containing a well-type NaI(Tl) scintillation detector. The detector was configured with a counting protocol specific to the ^99m^Tc gamma emission region of interest (corresponding to 140.57 keV (89 ± 4% intensity)). Samples were not analyzed until secular equilibrium between ^99^Mo and ^99m^Tc was attained (sample analyses were performed ∼24 h after each experiment was conducted). The *E*_d_ for the Wizard 1470 was determined by comparing the count rate of a 2.0 mL aliquot of ^99^Mo/^99m^Tc solution in the test tube *vs.* the 2.0 mL aliquot activity determined by the calibrated HPGe detector.

3. Benchtop NaI(Tl) detector/scaler: non-aqueous samples (*e.g.*, sample pan, trap components, furnace tube) were counted using a Ludlum 2200 scaler/rate meter coupled to a 2′′ dia. NaI(Tl) scintillation detector (Sweetwater, TX). Sample observation distance was maximized to ≥15 cm to minimize geometry effects. At a given sample/detector distance, *E*_d_ was determined by comparing the NaI(Tl) detector count rate with that of the HPGe-analyzed standard as described above. Again, samples were not analyzed until secular equilibrium between ^99^Mo and ^99m^Tc was attained.

### 2.6 Mass spectrometric measurements

After complete decay of ^99^Mo, dilutions of the dissolved Ni sample pan and trap leachates were prepared in 2% Optima grade HNO_3_. Quantification of U in the diluted solutions was performed by an Agilent 7700X (Ventura, CA) ICP-MS. Sample solutions were delivered to the mass spectrometer with a fluoride-resistant polyfluoroalkoxy alkane sample intake and nebulizer (Glass Expansion, Pocasset, MA). A ten-point calibration curve was prepared by gravimetric dilutions from a NIST traceable 1000 ppm single element U standard obtained from High Purity Standards (Charleston, SC). The calibration curve had a regression coefficient of 0.9999.

## 3. Results and discussion

### 3.1 Evaluation of Mo, Tc, and UO_2_ volatility by fluorination

**Fig. 2:**
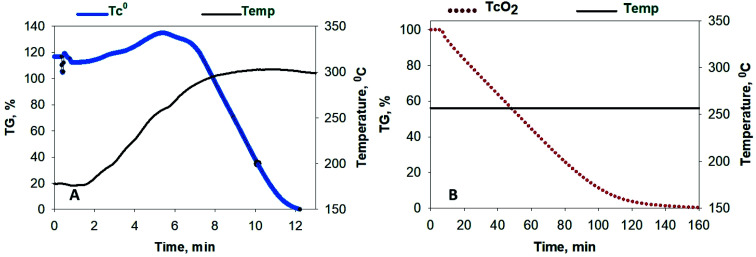
Conversion of ^99^Tc metal and ^99^TcO_2_ to their volatile fluorides (^99^TcF_6_) by exposure to 5% NF_3_ (in Ar). (A) Evolution of ^99^Tc metal, which initiates at ∼180 °C, and (B) ^99^TcO_2_, which initiates at or below ∼250 °C.

Fluoride volatility of Tc was likewise evaluated under the same conditions. Fig. 2A shows the evolution of ^99^TcF_6_ from ^99^Tc metal, which initiates at ∼180 °C, and that from ^99^TcO_2_, which initiates at or below ∼250 °C isothermal in Fig. 2B. The volatile reaction products are analogous to the Mo complexes discussed above. In Fig. 1B and 2A and B, the volatile species of Mo and Tc were removed from the reaction system by the Ar gas purge as demonstrated by the steep downward slopes of the TG scans. The Mo and Tc volatility profiles were found to be quite similar, and the complete removal of Te, Ru, Nb, Sb, and several other elements have been shown previously to follow suit.^17,18^

**Fig. 3:**
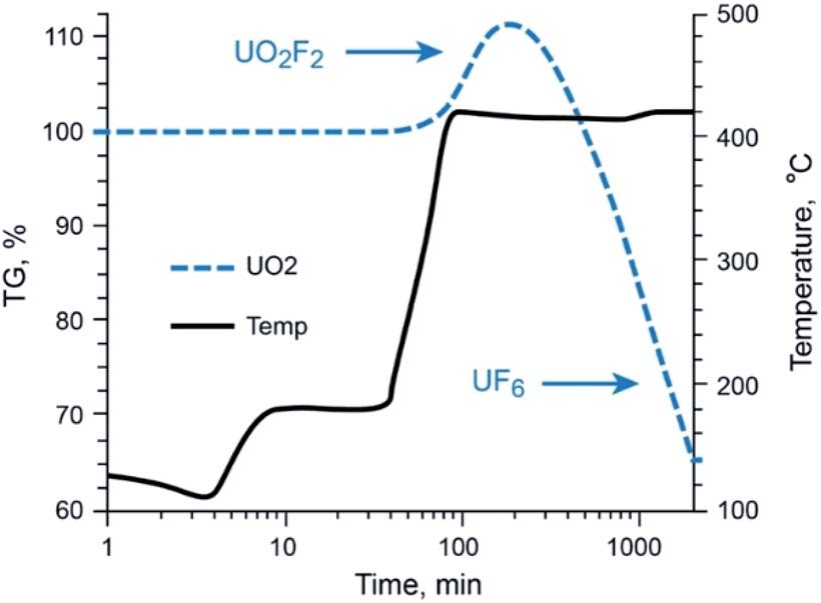
Thermo-fluorination conversion of UO_2_ to UO_2_F_2_ to UF_6(g)_ with 5% NF_3_. UO_2_F_2_ to UF_6(g)_ conversion occurs at temperatures well above that required for volatile MoF_6_/^99^TcF_6_ formation.

The behavior of UO_2_ with exposure to NF_3_ provides a stark contrast to that observed with Mo and Tc species, as shown in Fig. 3. Fluorination of UO_2_ is quite unique to this oxide of U and has been described previously by members of this research team.^12^ Using the same 5% NF_3_/Ar mixture employed for Mo- and Tc-bearing materials, UO_2_ was converted to non-volatile UO_2_F_2_ once the temperature approached 420 °C, after which a plateau region was sustained for several hours with the proper NF_3_ exposure conditions before significant production of gaseous UF_6_ occurred. The thermogravimetric evaluations with gas streams of heated 5% NF_3_/Ar indicate that gas-phase separations of Mo (metal and MoO_2_) and Tc (metal and TcO_2_) from UO_2_ is feasible.

Gaseous fluorides of these transition metals can be generated at temperatures below the conversion temperature of UO_2_ to UF_6_ (*via* UO_2_F_2_ formation). This permits NF_3_ leaching of a fissioned UO_2_ solid with no UF_6_ attendant in the gaseous Mo (Tc) phase.

### 3.2 Gas-phase separation of [^99^Mo]MoO_2_ from UO_2_

Given the preceding thermogravimetric results for metal and metal oxide constituents and UO_2_, a gas-phase separation of ^99^Mo (as MoO_2_) from UO_2_ was evaluated. A sample was prepared in a nickel sample pan that consisted of a homogeneous mixture of fine UO_2_ (23 mg) and MoO_2_ crystals (1.5 mg); NaBH_4_ was initially used to reduce an aqueous solution of Na_2_[^99^Mo]MoO_4_ to form a composite isotope solid of [^99^Mo]MoO_2_*via*eqn (5).^22^

5 Na_2_MoO_4_ + NaBH_4_ + 2H_2_O → NaBO_2_ + MoO_2_ + 2NaOH + 3H_2_

For this experiment, the outlet of the modified TG furnace tube was connected to tandem traps (A and B) that were packed with activated alumina. A third trap (C) was packed with a compact bundle of quartz wool (Fig. 1A). A fluorination experiment was performed with a 5% NF_3_/Ar mixture, and the furnace temperature held at ∼400 °C for 2 h. At the end of the experiment, the trapping system components were disconnected from the furnace tube, and each of the three traps was disconnected from each other. Next, each component in Fig. 1A was leached using a series of solvent washes. These washes included that of the furnace tube and each of the three traps. The nickel sample pan (and salt residues) was completely dissolved in nitric acid. The water in the bubbler trap was acidified and evaporated to near dryness. Each component and wash solution was analyzed by gamma counting (^99^Mo/^99m^Tc) and ICP-MS (U).

Analysis of the distribution of [^99^Mo]Mo and U revealed an excellent separation of the fission product from the simulated fissioned source material. The Mo was almost completely removed from the sample pan, with only 4% remaining (Table 2). Approximately 10% was deposited on the walls of the furnace tube, and 71% was captured in Trap A. Less than 1% of Mo was measured in Traps B, C, and the bubbler. In total, 86% of the Mo was accounted for in the assays of the trapping components. Of the Mo captured in Trap A, ∼70% was removed with a 5 mL H_2_O rinse (representing ∼50% of the total Mo pan deposit), and an additional 21% was recovered in two sequential washes with NaOH (Table 3). Within the three Trap A aqueous washes, ∼65% of the pan-deposited Mo was recovered.

**Table 2:** Distribution of molybdenum and uranium following thermo-fluorination of [^99^Mo]MoO_2_/UO_2_ mixture

Component a	Isotope & elemental distribution	
	[^99^Mo]Mo, %	U, %
Post-fluorinated pan	4.34	95.3 ± 3.4
Furnace tube	9.71	0.024 ± 0.001
Trap A	71.38	<0.001
Trap B	0.42	<0.001
Trap C	0.39	0.002 ± 0.001
Bubbler trap	0.01	0.118 ± 0.004
Total yield	86.26	95.5 ± 3.4

a See Fig. 1 for component locations.

**Table 3:** Distribution of [^99^Mo]Mo recovered from Traps A and B using a multi-step aqueous recovery method

Treatment	Reagent	Volume, mL	Trap A recovery^a^, %	Trap B recovery^b^, %
Elute 1	H_2_O	5	69.8	55.0
Elute 2	4 M NaOH	5	17.0	7.6^c^
Elute 3	4 M NaOH	5	4.0	—
Al_2_O_3_ leach	4 M NaOH, Δ^d^	5	5.7	23.5
Al_2_O_3_ residue	—	—	3.4	13.9

a Total recovered ^99^Mo activity fraction = 71.38% (from Table 2).

b Total recovered ^99^Mo activity fraction = 0.42% (from Table 2).

c Elutes 2 and 3 were combined into single vessel.

d Al_2_O_3_ in traps emptied into vessel followed by hot leaching with NaOH; leachate assayed for ^99^Mo activity.

Radiometric counting of the trapping components immediately after disassembly (before ^99^Mo/^99m^Tc secular equilibrium was attained) provided qualitative indication that ^99m^Tc was transported efficiently out of the pan and was successfully deposited primarily in the furnace tube and Trap A. Unfortunately, quantitative determination of the ^99m^Tc depositions were not possible with the use of the NaI(Tl) scintillation detector/scaler. However, an HPGe detector scan of the post-reacted Ni sample pan indicated that ^99m^Tc was successfully volatilized and transported out of the pan, thereby corroborating the observed volatilization profile shown in Fig. 2.

In sharp contrast, the U remained in a non-volatile state; 95.3 ± 3.4% of the original U deposit remained in the nickel sample pan, and ∼0.027% was found in the furnace tube (0.024%) and the three traps (0.003%, Table 2). Based on the mass of U measured in the combined trap leaches, the U decontamination factor in the Trap A [^99^Mo]Mo product was >1.0 × 10^5^. Total U recovery in all fractions was found to be 95.5 ± 3.2%, a value that was within the analytical uncertainty of the experiment.

## 4. Conclusions

We show that exposure of a homogeneous mixture of [^99^Mo]Mo/UO_2_ to 5% NF_3_/Ar mixture at ∼400 °C for ∼2 h results in a rapid, high yield extraction of [^99^Mo]Mo from U.

Of the ∼86% of ^99^Mo activity accounted for in the various furnace/trap components, ∼71% of the ^99^Mo activity was deposited in the first alumina trap. A simple 5 mL water wash of the trap's alumina bed resulted in ∼70% of the trapped ^99^Mo activity removal, which represented ∼50% of the total ^99^Mo activity originally deposited in the nickel pan. Technetium-99m was likewise transported and collected on the alumina trap with the separated ^99^Mo product, although quantitative distribution was not possible in this first test. The results indicate that the gas-phase [^99^Mo]Mo product was largely devoid of U contamination.

Aqueous processing releases I, Te, Xe and Kr potentially at every step of processing of irradiated targets. Acid dissolution, in particular promotes, volatile behavior in several elements as Tc, and Ru. While fluoride volatility must release these species as well, we believe that the front-end processing of irradiated uranium targets by volatility-based separations is better suited by its rigorous closed engineering to sequester radionuclide populations than the digest and back end, clean-up approach historically and currently used by most nuclear-related enterprises. Fluoride volatility separations of ^99^Mo from uranium, so described, has a sound chemical basis. Its practical implementation for radiopharmaceutical scale processing still requires elucidation of transport and capture technologies that are optimized for high efficiency retention of isotopes of pharmaceutical interest.

## Conflicts of interest

There are no conflicts to declare.

## Supplementary Material
